# Specific Protein Kinase C Isoforms α And βI Are Involved in Follicle-Stimulating Hormone-Induced Mouse Follicle-Enclosed Oocytes Meiotic Resumption

**DOI:** 10.1371/journal.pone.0045043

**Published:** 2012-09-18

**Authors:** Jianwei Wang, Qian Chen, Jinlian Zhou, Jing Wen, Fenghua Bian, Ge Li, Xinyi Mu, Yingying Han, Guoliang Xia, Meijia Zhang

**Affiliations:** 1 State Key Laboratory of Agrobiotechnology, College of Biological Sciences, China Agricultural University, Beijing, People’s Republic of China; 2 Department of Pathology, 306 Hospital of PLA, Beijing, People’s Republic of China; Institute of Zoology, Chinese Academy of Sciences, China

## Abstract

Protein kinase C (PKC) is involved in gonadotrophin-induced oocyte maturation. In the present study, we investigated the role of specific PKC isoforms in the process of follicle-stimulating hormone (FSH)-induced oocyte meiotic resumption. Small antral follicles (200–300 µm in diameter) were isolated from immature mice and cultured *in vitro*. FSH significantly induced follicle-enclosed oocytes (FEOs) meiotic resumption after 8 hr culture. However, the induced effect of FSH was dose-dependently inhibited by the specific PKC α and βI inhibitor Gö6976, and 100 nM Gö6976 completely blocked FSH function in oocyte meiotic resumption. Furthermore, FSH dramatically induced the expression of transcripts encoding epidermal growth factor (EGF)-like growth factors *Areg*, *Btc*, and *Ereg* mRNA levels, and up-regulated tyrosine phosphorylation level of EGF receptor (EGFR) in granulosa cells. Blocking the function of EGFR by AG1478 eliminated the effect of FSH-induced FEOs meiotic resumption, suggesting that FSH induced oocyte maturation through the activation of EGFR. FSH-induced phosphorylation of EGFR could also be inhibited by Gö6976. Next, we examined the effect of FSH on the expression and phosphorylation PKC α and βI. FSH induced the expression of PKC α at mRNA and protein level, and also up-regulated its phosphorylation level in granulosa cells after 8 hr culture. However, FSH had no effect on the expression of PKC βI but down-regulated its phosphorylation level. In conclusion, FSH-induced activation of PKC α alone, or together with the inactivation of PKC βI in granulosa cells, participates in mouse oocyte meiotic resumption, possibly by the activation of EGFR signaling pathway.

## Introduction

Fully-grown mammalian oocytes are maintained in meiotic prophase arrest until the preovulatory surge of luteinizing hormone (LH) triggers the resumption of meiosis and ovulation. Cyclic nucleotides cAMP and cGMP are crucial to the maintenance of meiotic arrest [1]–[3]. The preovulatory gonadotropin surge induces oocyte meiotic resumption by overcoming the environmental maintenance of meiotic arrest [1], via increasing epidermal growth factor (EGF)-like growth factors [4], and then the activation of mitogen-activated protein kinase (MAPK) 3/1 (ERK1/2) [5]–[6]. Recently, the results of several studies show that the activation of protein kinase C (PKC) is also involved in follicle-stimulating hormone (FSH) and LH-induced MAPK activation and c-Jun N-terminal kinase (JNK) phosphorylation in cumulus cells and oocyte meiotic resumption in many species [7]–[14] PKC activator, phorbol 12-myristate 13-acetate (PMA), induces cumulus-enclosed oocytes (CEOs) meiotic resumption, and PKC inhibitors, staurosporine, sphingosine, calphostin C, chelerythrine chloride and bisindolylmaleimide I, inhibit the effect of FSH on MAPK activation, activation of cyclin-dependent kinase 4 (CDK4), DNA synthesis and oocyte maturation [8], [12], [15]. Additional studies suggested that the LHR and FSHR could provoke twice increase in ERK1/2 phosphorylation at high densities and the delayed activation of ERK1/2 require PKC activation and inhibits aromatase [16].More recently, gonadotrophins (FSH + LH) could up-regulate tumor necrosis factor-α-converting enzyme (TACE) protein and protease activity in porcine CEOs [17], and PKC participates in gonadotrophins-induced oocyte maturation possibly by shed EGF and/or EGF-like factors [6].

PKC is described as one member of the families of serine/threonine kinases, which is the major intracellular kinase implicated in a large number of signal transduction processes transducing extracellular signals into intracellular events [18]. At least 11 isoforms have been identified and grouped into three subclasses based on cofactor requirements: classical (α, βI, βII, and γ), novel (δ, ε, θ, and η) and atypical (ζ, μ and λ/ι) PKCs. The classical PKC (cPKC) isoforms are activated by calcium and diacylglycerol in a phospholipid-dependent manner, while novel PKC (nPKC) isoforms activation is calcium-independent, and atypical PKC (aPKC) isoforms require neither calcium nor diacylglycerol [19]. Different PKC isoforms have been identified in mouse oocytes and cumulus cells [7], [20]–[22]. Classical PKCs (α, βI, β2, and γ), novel PKC δ, and atypical PKCs (λ/ι, μ, and ζ) are found to exist in mouse oocytes and PKCα, βI, γ, δ, ζ and ε are present in cumulus cells [7]. However, these PKC isoforms may have different roles during oocyte maturation, since stimulation of PKC is sufficient and necessary to cause the resumption of meiosis in CEOs, but blocks meiotic resumption of denuded oocytes [7], [23], [12]. Up to date, it is still not well known that the signal transduction process of specific PKC isoforms is involved in gonadotrophins-induced oocyte meiotic resumption.

In vitro cultured intact follicles, both LH and FSH have been observed to induce oocytes maturation [24]–[28].In the present study, we investigated the role of cPKCs in FSH-induced meiosis resumption in follicle-enclosed oocytes (FEOs) isolated from immature mice, since calcium/calmodulin-dependent protein kinase II is required for FSH-activated oocyte meiotic resumption [29].

## Materials and Methods

### Animal Treatment

Immature Kunming White mice (outbreed strain) aged 21-to 23-d-old with the body weight of 12–14 *g* were purchased from the Laboratory Animal Center of the Institute of Genetics (Beijing, China), and kept in the facility at China Agricultural University according to the guidelines for laboratory animals. All animal treatment procedures were approved by the Animal Care Committee of China Agricultural University (CAU) and all efforts were made to minimize suffering. Mice were housed under controlled temperature (23±2°C) and lighting (16 hr light/8 hr darkness) with food and water *ad libitum* in air–conditioned rooms. All experimental mice were killed by cervical dislocation.

### Chemicals and Culture Medium

All reagents were purchased from Sigma-Aldrich (St. Louis, MO, USA) unless otherwise specified. FSH was dissolved in 0.9% (w/v) saline solution to make stock solutions of 25 IU/mL. Gö6976 and AG1478 were dissolved in dimethylsulfoxide (DMSO) as 10 mM and 1 mM stock solutions, respectively. Epidermal growth factor (EGF. Biosource, Camarillo, CA, USA) and amphiregulin (AR. Biosource) were prepared with 1 mM and 1 mM, respectively. The stock solutions were kept at −20°C and were added to the culture medium before use.

The maturation medium used for follicle culture was minimum essential medium (GibcoBRL, Carlsbad, Cal,USA) with Earles’salt, supplemented with 75 µg/mL penicillin G, 50 µg/mL streptomycin sulfate, 0.23 mM pyruvate, 2 mM L-glutamine, 3 mg/mL bovine serum albumin, 5 µg/mL insulin, 5 µg/mL transferrin.

### Follicle Isolation and Culture

The ovaries were isolated from immature female mice, transferred to a 35 mm dish with 2 mL maturation medium. Follicles were isolated from the ovaries by careful manual dissecting under a microscope using a pair of 27 *g* needles, and small antral follicles (200–300 µm in diameter) without apparent sign of necrosis were selected for all the experiments [30]. The isolation process would last no more than 2 hr. Groups of 30–50 FEOs were transferred into a disc with 2 mL maturation medium. The maturation medium was equilibrated overnight before culture. The culture was carried out at 37°C in an atmosphere with 5% CO_2_ in air. At the end of culture, oocytes were liberated by manual rupture of the follicles using a pair of needles under an inverted microscope, and the meiotic statuses of the oocytes were examined and classified into germinal vesicle (GV; meiotic arrest), germinal vesicle breakdown (GVBD; meiotic resumption), and the first polar body (PB1; meiotic maturation). The percent of GVBD (including polar body 1) per total number of oocytes (% GVBD) were calculated. Oocytes that had degenerated were excluded.

In some experiments, culture medium was supplemented with FSH (0.05 IU/mL), Gö6976 (an inhibitor of PKC α and βI, 0.1µM), AG1478 (1 µM), EGF (10 ng/mL) and/or AR (100 ng/mL). Control medium was supplemented with same volume of saline solution or DMSO as compatible with the treatment group. For real-time PCR and western blotting, granulosa cells were isolated from FEOs cultured with different treatments. All samples were immediately frozen in liquid nitrogen and stored at −80°C until analyzed for gene or protein expression as described below.

### Real-time PCR Analysis

Total RNA was isolated and purified from frozen samples using the RNeasy micro-RNA isolation kit (Qiagen, Valencia, CA) according to the manufacturer’s instructions. Reverse transcription was carried out directly after RNA isolation using the QuantiTek reverse transcription system (Qiagen, Valencia, CA). Real-time PCR was then conducted to quantify the steady-state mRNA levels using an ABI 7500 real-time PCR instrument (Applied Biosystems, Foster City, CA). The results were first normalized to the expression levels of a housekeeping gene, ribosomal protein L19 (*Rpl19*), by the 2^-ΔΔCt^ method [31], and the expression levels of transcripts are presented as the ratio of treated groups to controls. PCR primer sequences for *Prckα* were 5′*-*TCCAGTGCCAAGTTTGCTGTT-3′ (forward) and 5′-TCGTCAGTGTCAGGTCCCTTATC-3′ (reverse). *Areg*, *Btc*, *Ereg* and *Rpl19* primers were reported previously [32]. To avoid false positive signals, dissociation-curve analyses were performed at the end of the amplification and the PCR products were applied to agarose gel electrophoresis to confirm the sizes. Moreover, the PCR products were purified and sequenced to verify sequence identity. The reactions were conducted at least in duplicate.

### Protein Extraction and Western Blotting

Granulosa cells were collected from 50 follicles for western blotting analysis. Proteins from granulosa cells were extracted with double-strength electrophoresis sample buffer after culture, supplemented with 1 mM phenylmethylsulfonylfluoride and 1 mM sodium orthovanadate for 20 min on ice, and stored at −80°C. Before electrophoresis, the protein samples were were boiled in water for 10 min, cooled down on ice immediately, and then centrifuged at 8,000*g* for 5 min. Then the protein samples separated on a 15% (w/v) SDS-PAGE resolving gel with 5% (w/v) condensed gel and transferred electrophoretically to a nitrocellulose membrane (Protran; Schleicher Schuell UK Ltd, London). Membranes were blocked in TBST (20 mM Tris-HCl, 150 mM NaCl and 0.05% Tween 20; pH 7.5) buffer containing 5% nonfat milk for 2 hr with shaking at room temperature followed by incubation with primary antibodies, rabbit anti-PKC α (Y124, Abcam, Cambridge, MA, USA. 1∶1000), rabbit anti-phospho-PKC α (phospho-T497, Abcam. 1∶5000), rabbit anti-PKC βI (C-16, Santa Cruz, CA, USA. 1∶500), rabbit anti-phospho-PKC βI (phospho-Thr 642, Santa Cruz. 1∶200), goat anti-phospho-EGF receptor (phospho-Tyr1173, Santa Cruz. 1∶300) in TBST buffer overnight at 4°C and then washed with TBST buffer. Membranes were then incubated with HRP-conjugated anti-rabbit or anti-goat secondary antibody (1∶5000) for 1 hr at room temperature and washed with TBST buffer. Blots were immersed for 1 min in enhanced chemiluminescence detection reagent (Pierce Biotechnology, Rockford, IL, USA), and then exposed to film. Immunoreaction signals were analyzed using gel-pro Analyzer 4.0. Each experiment was repeated at least three times.

### Statistical Analysis

All experiments were performed at least three times, and results were expressed as mean ± S.E.M. All proportional data were subjected to an arcsine transformation and analyzed using least squares ranges procedure by applying SAS software. Statistical significance was defined as *P*<0.05.

## Results

### The Effect of Specific PKC α and βI Inhibitor Gö6976 on FSH-induced Mouse FEOs Meiotic Resumption

**Figure 1:**
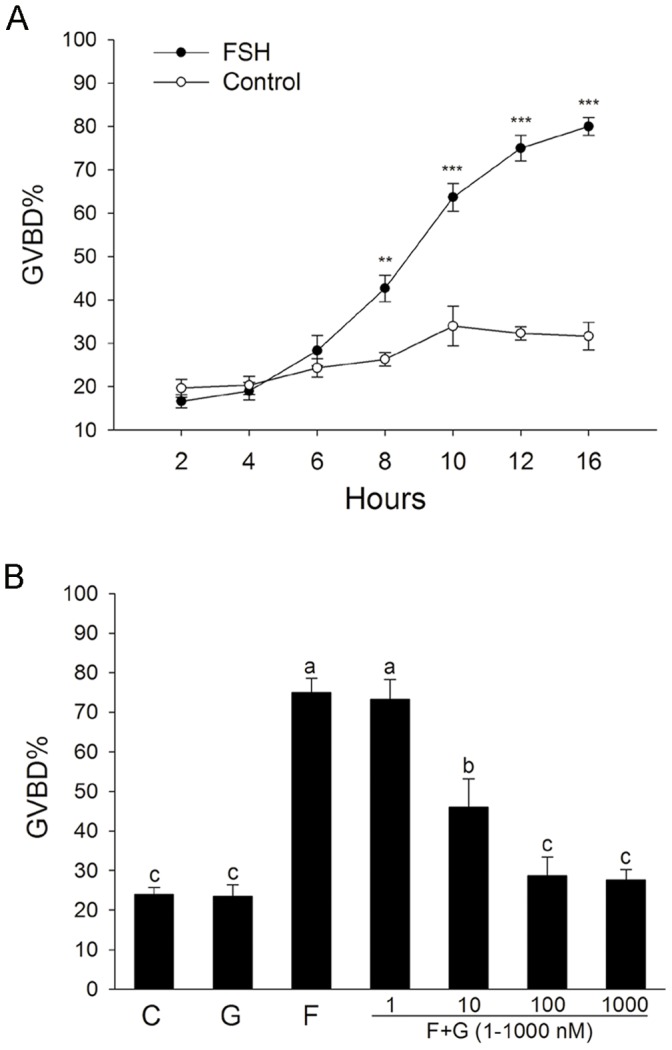
The effect of PKC α and βI inhibitor Gö6976 on FSH-induced mouse FEOs meiotic resumption. FEOs were cultured in MEM medium only, supplemented with FSH (0.05 IU/mL) and/or PKC α and βI inhibitor Gö6976 (1–1000 nM) for the indicated period. The percent of GVBD (including polar body 1) per total number of oocytes (% GVBD) were calculated. Oocytes that had degenerated were excluded. The similar demonstrations of statistical analysis were also used in the following Figures. Data were represented as mean percentage of GVBD ± SEM of at least three independent experiments. The median number of FEOs in each group was 150. (**A**) The kinetics of FSH-induced FEOs meiotic resumption. **P<0.01 and ***P<0.001 compared with each corresponding time point in the control groups. (**B**) Gö6976 dose-dependently inhibited the function of FSH. Groups with a different letter are significantly different (*P*<0.05). G, Gö6976; F, FSH.

As shown in Figure 1A, FSH significantly induced FEOs meiotic resumption after 8 hr (42.1%), 10 hr (62.9%), 12 hr (73.5%) and 16 hr culture (79.4%) compared with control (8 hr, 26.5% GVBD; 10 hr, 34.5% GVBD; 12 hr, 32.4% GVBD; 16 hr, 32.3% GVBD), consistent with our previous study [29]. However, FSH-induced FEOs meiotic resumption was dose-dependently inhibited by specific PKC α and βI inhibitor Gö6976, and 100 nM Gö6976 completely blocked the function of FSH on oocyte meiotic resumption (Figure 1B). Gö6976 (100 nM) alone had no effect on the resumption of meiosis in FEOs.

### The Effect of FSH on the Expression of Transcripts Encoding EGF-like Growth Factors

**Figure 2:**
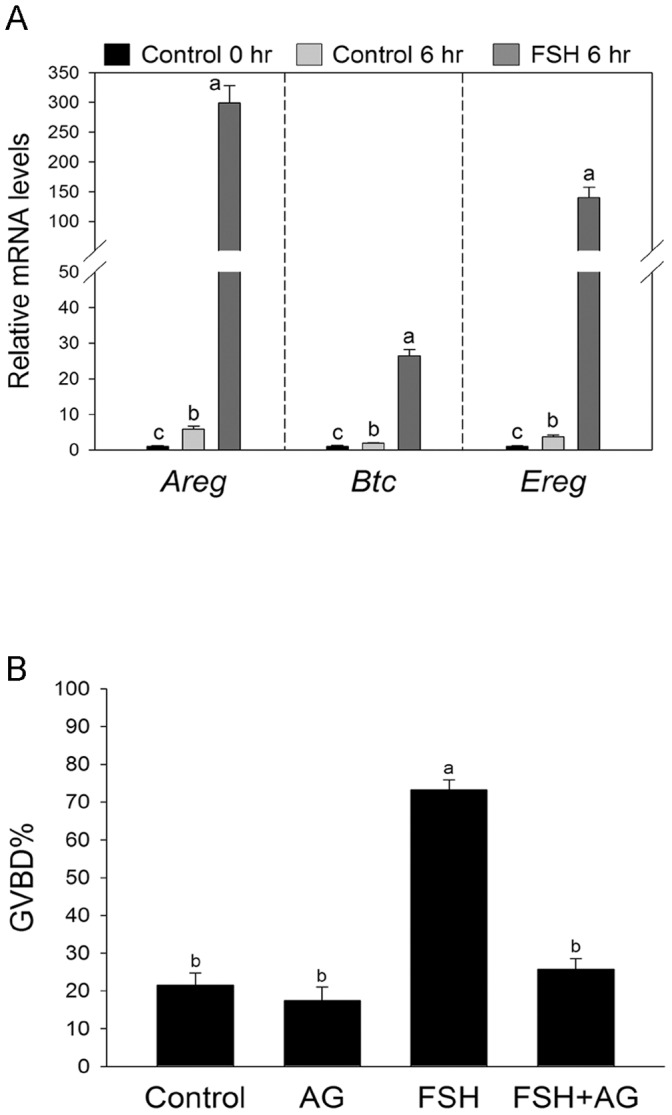
The effect of AG1478 on FSH-induced mouse FEOs meiotic resumption. (**A**) FSH promoted the expression of transcripts encoding EGF-like growth factors *Areg*, *Btc*, and *Ereg* mRNA. Follicles were cultured in MEM medium with or without FSH, and granulosa cells were collected at the indicated culture time. The mRNA level of *Areg*, *Btc*, and *Ereg* was normalized to the level of *Rpl19*. Mean value of granulosa cells at 0 h was normalized to 1. Bars show the mean ± SEM of three independent experiments. Relative levels with a different letter are significantly different (*P*<0.05). (**B**) FEOs were cultured in MEM medium supplemented with 0.05 IU/mL FSH and/or 1 µM AG1478 for 16 h. Data were represented as mean percentage of GVBD ± SEM of four independent experiments. The median number of FEOs in each group was 170. Groups with a different letter are significantly different (*P*<0.05). AG, AG1478.

**Figure 3:**
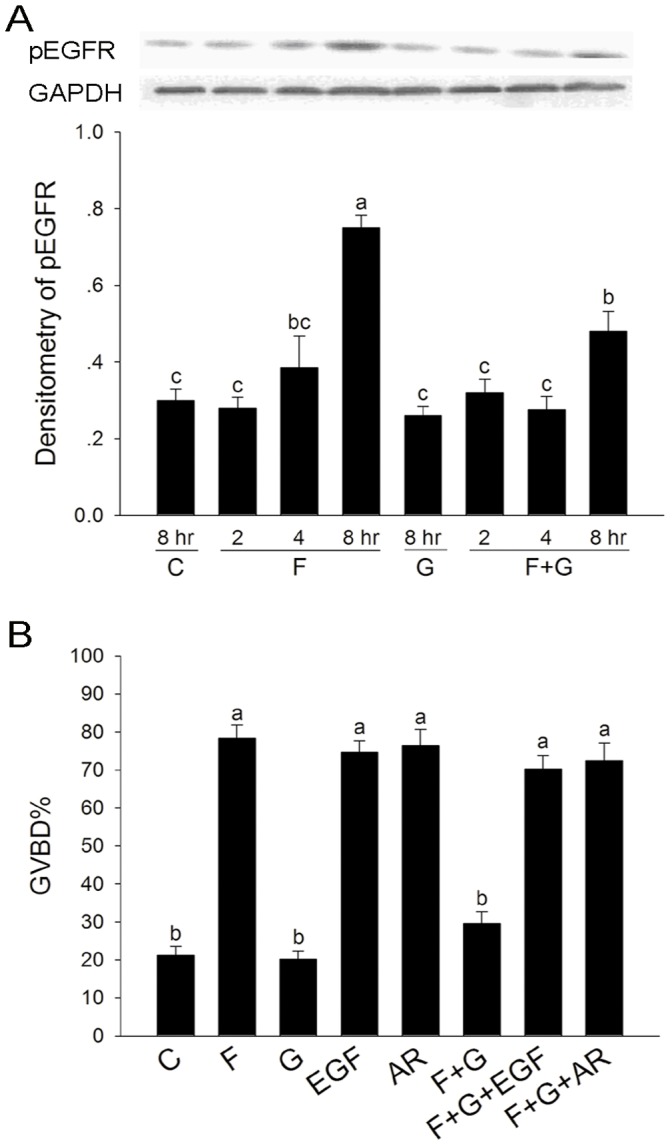
The effect of Gö6976 on the phosphorylation of EGFR during FSH-induced FEOs meiotic resumption. (**A**) Granulosa cells were collected at the indicated culture time, and analyzed by western blotting. Immunoblots were performed by phospho-EGF receptor antibody, and GADPH was served as an internal standard. Relative intensities were quantified using Gel-pro analyzer 4.0 software. Bars show the mean ± SEM of three independent experiments. Relative levels with a different letter are significantly different (*P*<0.05). (**B**) FEOs were cultured in MEM medium supplemented with 0.05 IU/mL FSH, 0.1 µM Gö6976, 10 ng/mL EGF and or 100 ng/mL AR for 16 h, and the rate of GVBD was scored at the end of culture. Data were represented as mean percentage of GVBD ± SEM of three independent experiments. The median number of FEOs in each group was 120. Groups with a different letter are significantly different (*P*<0.05). C, control; F, FSH; G, Gö6976.

It is reported that EGFR activation participates in FSH-induced oocyte meiotic resumption when cumulus oocyte complexes were used [33]–[34]. We examined the effect of EGFR activation on FSH-induced meiotic resumption of cultured follicle-enclosed mouse oocytes. During the FEOs culture, the expression of transcripts encoding EGF-like growth factors *Areg*, *Btc*, and *Ereg* mRNA levels was increased in granulosa cells, but dramatically increased after FSH treatment (Figure 2A). FSH also significantly increased phosphorylation level of EGFR (Figure 3A). In order to examine whether these EGF-like growth factors were involved in FSH-induced FEOs meiotic resumption, the special inhibitor of EGF receptor (EGFR) AG1478 was added to the medium. As shown in Figure 2B, 1 µM AG1478 completely reversed FSH-induced FEOs meiotic resumption, suggesting that FSH, via EGFR pathway, induced the meiotic resumption of FEOs.

### The Effect of Gö6976 on FSH-induced the Phosphorylation of EGFR

We examined the effect of PKC α and βI inhibitor Gö6976 on FSH-induced EGFR activation, since FSH induced FEOs maturation through the activation of EGFR. As shown in Figure 3A, FSH significantly induced tyrosine phosphorylation of EGFR in granulosa cells at 8 hr culture, which was inhibited by Gö6976. As the activators of EGFR, both EGF and Amphiregulin (AR) could induce oocytes maturation, and also reverse the inhibitory effect of Gö6976 on the action of FSH (Figure 3B). These results suggested that PKC **α** and βI participated in FSH-induced FEOs meiotic resumption probably by the activiation of EGFR.

### The Effect of FSH on the Expression and Phosphorylation of PKC α

**Figure 4:**
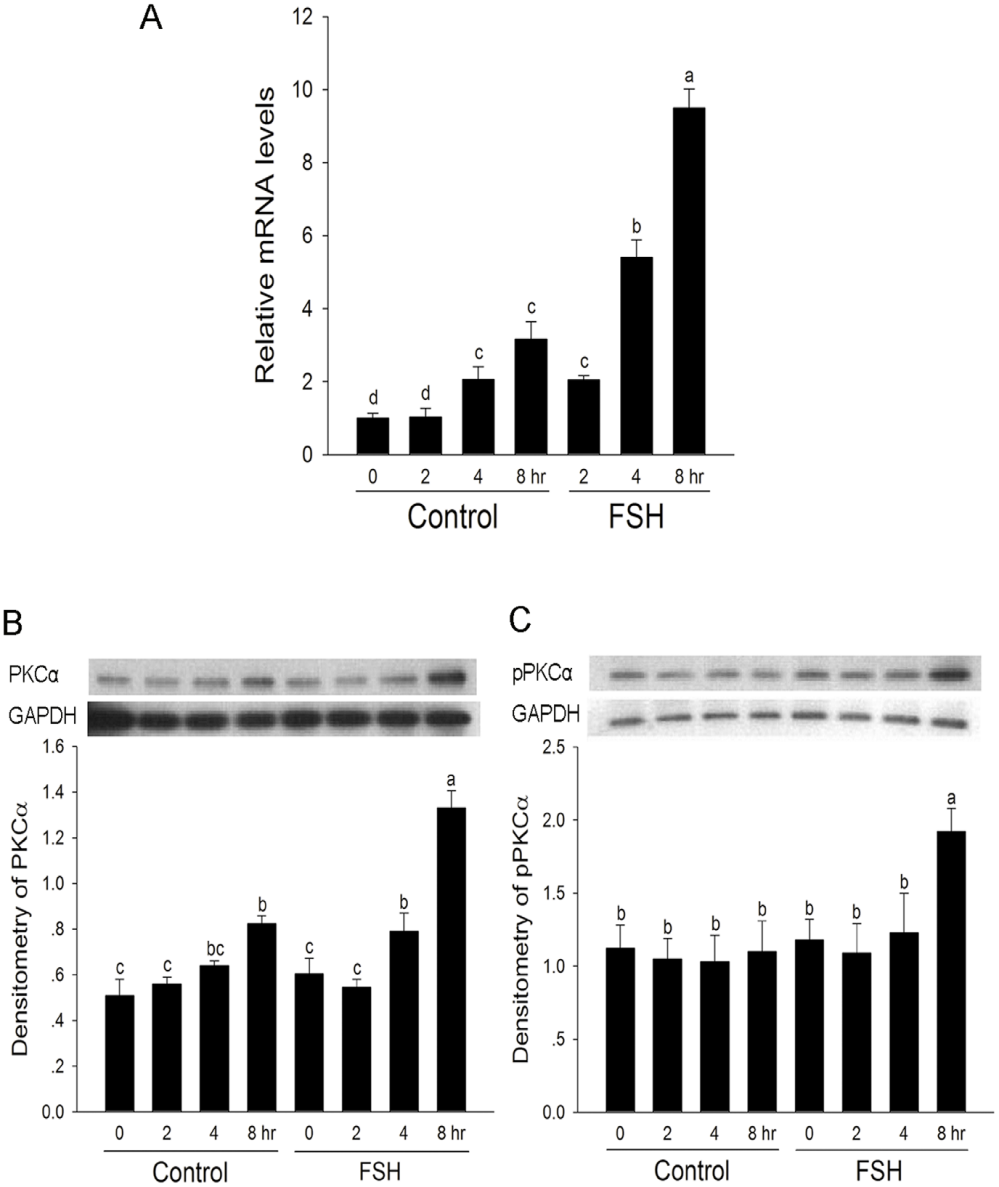
The effect of FSH on the expression and phosphorylation of PKCα. Follicles were cultured in MEM medium with or without FSH, and granulosa cells were collected at the indicated culture time. (**A**) FSH promoted the expression of *Prckα* mRNA. The mRNA level of *Prckα* was normalized to the level of *Rpl19*. Mean value of granulosa cells at 0 h was normalized to 1. The values indicated by different letters are significantly different (*P*<0.05). (**B**) and (**C**) FSH promoted the expression and phosphorylation of PKCα. In each treatment, granulosa cells from 50 follicles cultured were lysated, separated by 15% SDS-PAGE, and analyzed by western blotting. Immunoblots were performed by PKCα antibody and PKCα-phospho-specific Tyr497 antibody, respectively. GADPH was served as an internal standard. Relative intensities were quantified using Gel-pro analyzer 4.0 software. Bars show the mean ± SEM of three independent experiments. Relative levels with a different letter are significantly different (*P*<0.05).

The effect of FSH on the mRNA level of *Prckα* in granulosa cells was determined by QRT-PCR analysis. As shown in Figure 4A, the levels of *Prckα* mRNA were gradually increased during the culture period. FSH significantly induced the steady-state level of *Prckα* mRNA at 2, 4 and 8 hr compared with control. Western blotting showed that the level of PKC α protein was increased after 8 hr of culture, but dramatically increased after FSH treatment (Figure 4B). At the same time, FSH significantly increased the phosphorylation level of PKCα at 8 hr compared with control (Figure 4C).

### The Effect of FSH on the Expression and Phosphorylation of PKC βI

**Figure 5:**
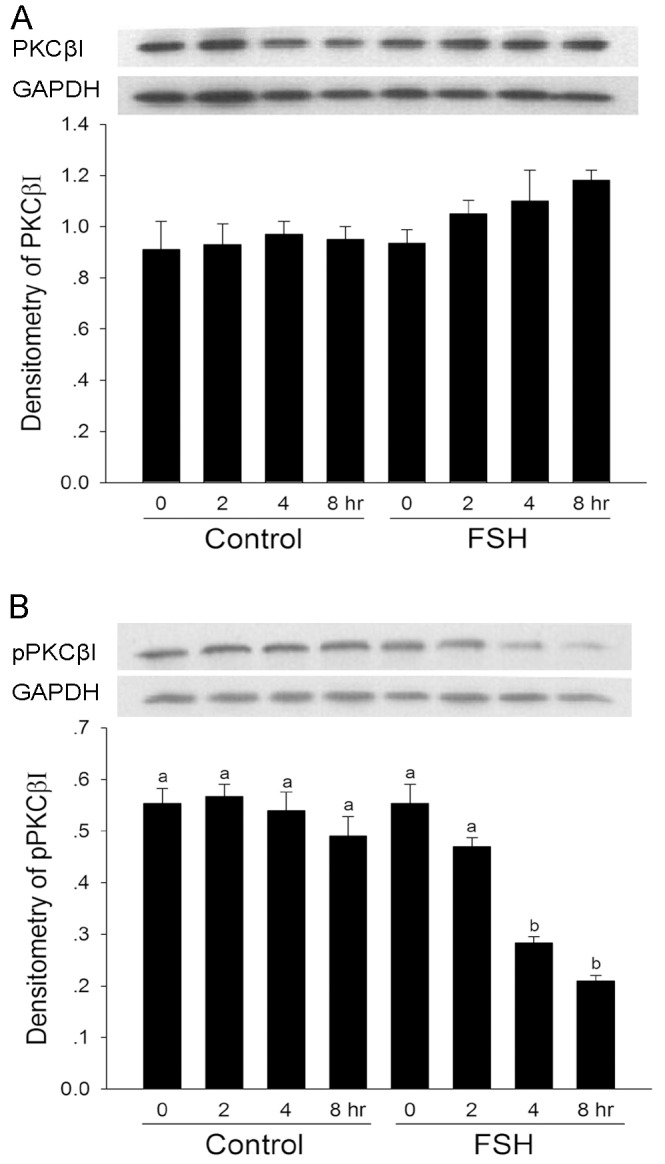
The effect of FSH on the expression and phosphorylation of PKC βI. Follicles were cultured in MEM medium with or without FSH, and granulosa cells were collected at the indicated culture time. Immunoblots were performed by PKC βI (**A**) and PKCβI-phospho-specific Thr 642 (**B**) antibodies, respectively. GADPH was served as an internal standard. Relative intensities were quantified using Gel-pro analyzer 4.0 software. Bars show the mean ± SEM of three independent experiments. Relative levels with a different letter are significantly different (*P*<0.05).

The effect of FSH on the expression and phosphorylation of PKC βI protein in granulosa cells was determined by western blotting. As shown in Figure 5A, the levels of PKC βI protein in granulosa cells were not obvious change during the culture period, and FSH also had no effect on the expression of PKC βI protein. However, FSH significantly decreased the phosphorylation level of PKC βI at 4 and 8 hr compared with control (Figure 5B).

## Discussion

In the present study, we demonstrated the role of cPKC isoforms on FSH-induced oocyte meiotic resumption with a follicle culture model. FSH induced the expression of PKC α at mRNA and protein level, and the phosphorylation of PKC α in granulosa cells. However, FSH had no effect on the expression of PKC βI translation, but down-regulated its phosphorylation level. The inhibitor of PKC α and βI, Gö6976, completely blocked FSH-induced FEOs meiotic resumption. Furthermore, FSH dramatically induced the expression of transcripts encoding EGF-like growth factors *Areg*, *Btc*, and *Ereg* mRNA levels, and the phosphorylation of EGFR in granulosa cells, which participated in FSH-induced FEOs meiotic resumption. FSH-induced phosphorylation level of EGFR could be inhibited by Gö6976. All these results suggest that cPKC isoforms α and βI participate in FSH-induced mouse FEOs meiotic resumption, possibly by the activation of EGFR.

It is reported that the activation of PKC is involved in FSH -induced mitogen-activated protein kinase (MAPK) activation in cumulus cells and oocyte meiotic resumption in many species [7]–[10], [12]. Furthermore, the specific EGF receptor kinase inhibitor AG1478 could block both FSH and PMA-induced porcine and mouse COCs meiotic resumption [33]–[34], suggesting that PKC may regulate the action of FSH by activating EGFR and then MAPK signaling pathway. In our study, FSH induced the expression of transcripts encoding EGF-like growth factors *Areg*, *Btc*, and *Ereg* mRNA, and the activation of EGFR in granulosa cells. Furthermore, the inhibitor of EGFR AG1478 completely blocked FSH-induced FEOs meiotic resumption, consistent with the previous studies that FSH induces CEOs meiotic resumption via the activation of EGFR [33]–[34].The specific PKC α and βI inhibitor Gö6976 inhibited FSH-induced phosphorylation level of EGFR, suggesting that FSH activates EGFR signaling pathway partly via PKC α and βI. These results are consistent with previous reports that PKC mediates the action of FSH by activating EGFR signaling pathway [33]–[34]. The inhibitory effects of Gö6976 on FSH-induced meiosis resumption could be reversed by EGF and AR, indicating that PKC α and βI participate in FSH-induced FEOs meiotic resumption possibly by shed EGF and/or EGF-like factors [6].

Further, it is concluded that EGF-like factors released from granulose cells diffuse to other granulosa cells within the follicle to act in paracrine/autocrine fashion, to stimulate ERK1/2MAPK-dependent gene transcription in cumulus cells, including induction of prostaglandin synthase 2 and increased PGE2 production that mediates later stages of ovulation. The downstream positive signal that triggers GVBD remains unknown, but appears to be transmitted directly from cumulus cells to the oocyte through gap junctions coupling these two cell types [34]–[35]. However, PKC pathway seems to be essential for this process. Downs’ study has suggested that direct activation of PKC in the oocyte suppresses maturation, while stimulation within cumulus cells generates a positive trigger that leads to meiotic resumption. Activation of PKC in the granulosa cells stimulates the production of a positive activator (Act) that traverses the gap junctions coupling the somatic and germ cell compartments to trigger GVBD in the oocyte. It is also possible that an activator is secreted and contributes to meiotic induction in a paracrine fashion [7].

In the present study, FSH induced the expression and phosphorylation of PKC α in FSH-induced FEOs meiotic resumption, which suggested that PKC α participated in FSH-induced FEOs meiotic resumption. However, FSH also decreased the phosphorylation level of PKC βI but had no effect on the expression of PKC βI in FSH-induced FEOs meiotic resumption. It is possible that FSH-induced dephosphorylation of PKC βI may also participate in FSH-induced oocyte maturation. However, we could not study the individual role of PKC α and PKC βI, respectively, because of the lack of PKC α or PKC βI specific inhibitor. FSH-induced FEOs meiotic resumption could be completely blocked by the inhibitor of PKC α and βI, Gö6976. The inhibitory effect of Gö6976 may be mainly via inhibiting the function of PKC α, since PKC βI is poorly involved in FSH-induced the meiotic resumption of COCs [36]. The inhibition of PKC βI by G ö6976 and LY333531 (PKC βI and βII specific inhibitor) could not induce oocyte maturation (Fig. 1B and data not shown), indicating that the inhibition of PKC βI may have no effect on the stimulation of oocyte maturation. The classical PKCs are activated by calcium in a phospholipid-dependent manner, and these calcium-dependent pathways are essential for FSH-induced oocyte meiotic resumption of mouse oocytes [37]. Further study is needed to elucidate whether Ca^2+^ is involved in the activation of PKC α and/or PKC βI during FSH-induced oocytes maturation.

Recently, the specific functions and the spatio-temporal distributions of PKC isoforms especially the cPKCs have been studied on the oocyte maturation. The PKC activator phorbol esters (TPA) induces depletion of PKC α from the cytoplasm followed by the accumulation of this isoform at the plasma membrane, and results in the spontaneous GVBD inhibition in mouse CEOs [38]. However, other studies show that the translocation of PKC α and PKC β from the cytoplasm to the nuclus was necessary for oocyte meiosis resumption [39]. The different functions of PKC α and β may be due to the different culture models and the way that the oocytes are treated. These studies implied that the specific isoforms of PKCs in oocyte are activated during oocyte maturation. In our study, we found that FSH up-regulated the phosporylation level of PKC α, but down-regulated the phosporylation level of PKC βI in granulosa cells, by which FSH probably induced FEOs maturation. Further work is needed to examine the effect of FSH on phosporylation and translocation PKC α and βI in oocyte.

Although FSH and LH stimulate distinct physiological responses [40], several studies have indicated that both of the two gonadotropins use EGFR signaling as a potential central pathway in murine oocyte maturation [4], [33]–[34]. These findings suggest that the mechanism of FSH- and LH-induced oocyte maturation may share some similarities. The study using small antral follicle culture model may help us comprehend the potential signal pathways in gonadotropin-induced oocyte maturation. In the present study, FSH induced the expression and phosphorylation of PKCα, but decreased the phosphorylation level of PKC βI in FSH-induced meiotic resumption of cultured follicle-enclosed mouse oocytes. Furthermore, FSH endogenously stimulated EGFR activators expression, which activated EGFR signaling pathway and then FEOs meiotic resumption. The PKC α and βI inhibitor Gö6976 inhibited FSH-induced EGFR activation and oocyte maturation, but EGF and AR could reverse the inhibitory effect of Gö6976 on the action of FSH. Together, this study revealed that PKC specific isoforms α and βI are involved in FSH-induced mouse FEOs meiotic resumption, possibly by the activation of EGFR.
